# Prevalence of group a streptococcus pharyngeal carriage and clinical manifestations in school children aged 5–15 yrs in Wakiso District, Uganda

**DOI:** 10.1186/s12879-017-2353-5

**Published:** 2017-04-05

**Authors:** Irene Nayiga, Emmy Okello, Peter Lwabi, Grace Ndeezi

**Affiliations:** 1 Department of Paediatrics & Child Health, College of health sciences, Makerere University, P.O Box 7072, Kampala, Uganda; 2 Uganda Heart Institute, Mulago Hospital Complex, PO Box 7051, Kampala, Uganda

**Keywords:** Group a streptococcus, Carriage, Uganda

## Abstract

**Background:**

Beta-hemolytic streptococci carrier rates in children living in low-income countries are high ranging from 10 to 50%. Although most of these children are asymptomatic, they are a reservoir and pose a risk of transmission. The aim of this study was to determine the prevalence of group a streptococcus pharyngeal carriage and clinical manifestations in school going children in Wakiso district, Uganda.

**Methods:**

A cross sectional study targeting children age 5–15 years in primary schools in one sub-county of Wakiso district was carried out. Three hundred and sixty-six children from five primary schools were enrolled and evaluated for group a streptococcus (GAS) carriage. A semi-structured questionnaire was used to collect data that included social demographics, school environment and clinical findings. For every enrolled child a throat swab was taken and cultured for GAS and blood was drawn for anti-streptolysin-O titres. Analysis of data was done using STATA.

**Results:**

The prevalence of GAS carriage was 16%. The children with GAS positive cultures were mainly females. The factor associated with GAS carriage was the school location, with peri-urban schools more likely to have children with GAS compared to rural schools; AOR 2.48 (95% CI: 1.01 – 6.11), *P* = 0.049. There was no significant difference between the characteristic of children with GAS positive verses GAS negative throat swab cultures.

**Conclusion:**

There is a high prevalence of GAS pharyngeal carriage among children aged 5–15 years attending primary schools in Wakiso District, Uganda.

**Electronic supplementary material:**

The online version of this article (doi:10.1186/s12879-017-2353-5) contains supplementary material, which is available to authorized users.

## Background

Group a beta-hemolytic streptococcus (GAS) has been estimated to account for between 20 to 40% of all cases of pharyngitis in children [1]. There are 450 million estimated cases of GAS pharyngitis in children each year worldwide [2]. GAS pharyngitis is ubiquitous but is more frequent in low-income countries. Surveys done in healthy school children in the age range of 6 to 10 years found anti-streptolysin-O titers of more than 200 Todd units in 15 to 70% of the children [3] while other studies have reported beta-hemolytic streptococci carrier rates of 10 to 50% for asymptomatic school children [4].

Group a streptococcus, the most common bacterial cause of pharyngitis, has a peak incidence in children 5–15 years of age [5]. Pharyngitis caused by group a streptococcus has been linked with the aetiopathogenesis of rheumatic fever (RF) and rheumatic heart disease (RHD) [5]. Primary prevention relies on the eradication of group a streptococcal carriage through active sore throat screening and by treatment of pharyngitis with oral antibiotics [6].

The current estimated prevalence of RHD in Kampala, Uganda is 14 per 1000 cases among primary school children and this is a sequeale of group a streptococcus pharyngitis [7]. The prevalence of group a streptococcus in school children in Uganda had not been described, despite having several studies done regarding the most adverse sequeale i.e. RF and RHD.

The aims of this study were to determine the prevalence of GAS pharyngeal carriage, describe the clinical characteristics of the children with GAS and determine factors associated with GAS pharyngeal carriage in school going children in Sissa sub-county, Wakiso district, Uganda.

## Methods

This was a cross sectional study targeting children between the ages of 5 to 15 years that were enrolled in primary day schools in Sissa sub-county, Wakiso district. Wakiso district is in the central region of Uganda. It forms a crescent like boundary that almost surrounds Kampala, the capital city of Uganda.

Wakiso is the most populated district in Uganda with 2,007,700 people. The average household size in this district is 3.9 people. Sissa sub- county lies in the southern part of Wakiso and has a population of 93,238 people.

Children age 5–15 yr. attending primary day schools in Sissa Sub-county, Wakiso district whose parents gave informed consent were enrolled in this study. In addition, assent was attained from pupils aged >8 yrs. The children who were on antibiotic treatment during the study period were excluded. Determination of antibiotic use was by self-report at the time of enrollment.

A multi-stage sampling design was employed for this study. Sissa sub-county was randomly selected from the eight sub-counties of Wakiso district with peri-urban and rural populations. Five primary schools Kitende, Bethel, Mpumudde, Jjanyi and Faith Trust were then randomly selected from the list of schools in the sub-county. Using class registers and the assistance of the teachers, eligible children were selected based on the number of children in each class. Every third child starting with the first was selected to participate in the study. The head teachers and school contact persons were informed that any child whose throat swab culture turned positive would be informed and referred to the nearest health center for treatment.

Based on a study by Shaikh N et al. [1] the GAS carriage prevalence of 12% was used to calculate the sample size of 325 pupils using the Leslie Kish formula. The sample size for the factors associated was 291 by Hsieh formula. The larger sample size was considered and 10% was added for non-response.

Asymptomatic GAS pharyngeal carriage was defined as a positive throat culture for GAS without a GAS specific immune response [8]. The immune response considered in this study was antistreptolysin-o antibody titre (ASOT).

Normal values of ASOT are variable and usually depend on the age of the patient, geographical location, epidemiological settings and season of the year [4]. An ASOT level of more than 200 Todd units was taken as generally increased since there was no documentation of the normal values in our setting.

The study examined the school environment such as location [rural, peri-urban], school ownership, number of pupils per class and class floor area. The socio-demographics including age, sex, number of children in the class, number of people at home (household size), type of housing and parent’s occupation were recorded for each child. History of fever, sore throat, cough plus documentation of RHD were recorded.

The principal investigator and a medical officer did general exam of the children. This included measuring weight, height, temperature, examination of cervical lymph nodes and the throat. Weights were taken to the nearest 100 g using a SECA scale. The children were weighed with minimum clothing and standing height was taken using a stadiometer. A body mass index (BMI) was then calculated. A throat swab and blood sample was taken from every child enrolled in the study.

Throat swab collection: In good light, the children opened their mouths as wide as possible. Using a tongue depressor, the investigator looked for inflammation and presence of any exudates or pus in tonsillar area. With a sterile cotton swab, the investigator rubbed on back of the throat and on the tonsils. The swab was then placed in an Amies transport media container, labeled with the study ID number. The sample was placed in a biohazard bag and delivered to the laboratory.

Blood collection for anti-streptolysin O titres (ASOT). The skin around the cubital fossa was cleaned using 70% isopropyl alcohol. 3mls of blood was drawn and collected in plain serum (red) top vacutainers.

At the end of the recruitment day, the specimens were transported to a certified clinical laboratory in Kampala for analysis. At the laboratory, the throat swabs were inoculated on blood agar medium. A bacitracin disk was placed in the growth media and incubation was done at 35–37 °C in an incubator. After 24–48 h, plates were examined for tiny colonies of about 0.5 mm diameter, with a wider zone of hemolysis. These colonies were sub-cultured on a new plate of blood agar and susceptibility testing was done.

Susceptibility testing was conducted using the disc diffusion method on Mueller Hinton agar and the antibiotic panel included penicillin, ceftriaxone, vancomycin, erythromycin, azithromycin, tetracycline and chloramphenicol.

A rapid latex agglutination test for the qualitative determination of anti-streptolysin-O (ASO) antibodies in serum was used. A negative result had no agglutination of the latex particles suspension and a positive result had an agglutination of the latex particles suspension, indicating an ASO level of more than 200 IU/ml.

In the laboratory, qualified laboratory technicians used standard operating procedures to process the samples and weekly quality control tests were done.

Analysis of data was done using STATA. At the univariate level, means, medians and their measures of dispersion were used to describe continuous variables. Proportions were used to describe categorical variables and the prevalence of GAS carriage. At the bivariate and multivariate analysis, unadjusted and adjusted Odds ratios were used to determine the factors associated with GAS pharyngeal carriage.

### Ethical approval

Permission to carry out the study was sought from School of Medicine Research and Ethics Committee, College of Health Sciences Makerere University, Wakiso district education department, heads of school, parents and children aged 8 years and above. Children with GAS positive cultures received a 10-day course of amoxicillin at government health centers. Amoxicillin is on the essential drug list that the ministry of health Uganda provides to all health facilities.

## Results

**Fig. 1:**
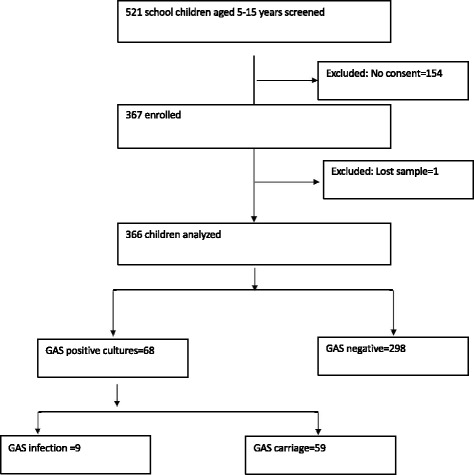
Study profile

Three hundred sixty-six children, aged 5–15 years took part in this study. Enrollment of the study participants took place between 21 July and 29 October 2014 (Fig. 1). The five primary schools selected were Kitende, Bethel, Mpumudde, Jjanyi and Faith trust primary school. Three out of the five are government-aided schools. Kitende, Bethel and Faith trust are peri-urban schools. Bethel primary school has 20% of its pupils in the day section. The average number of children per square of classroom floor area was 1.3 within the enrolled schools. Table 1 shows the proportions of the pupils enrolled per school in the study.

**Table 1:** School enrolment and classroom space per child in five selected schools in Sissa sub-county, Wakiso

School	Total class floor area(m^2^)	Total school enrollment	Number of children per sq. meter of floor area	Number enrolled in the study	Proportion enrolled in the study (%)
Kitende primary	331.21	554	1.6	119	32
Bethel primary	446.89	510	1.14	58	16
Mpumudde primary	314.87	228	0.72	54	15
Jjanyi primary	376.62	270	0.72	72	20
Faith trust primary	269.99	392	1.45	64	17

Of the 366 children, 36.3% were below 10 years while the rest above 10 years of age. Their mean age was 10 yrs. (SD 2.6). The female children were 217/366 (59.3%) of the enrolled population. The average household was six people (SD 2.5). Out of the 366 pupils, 241(66%) were from peri-urban schools. For all the schools the average class size was 63 children (SD 26). Table 2 shows demographic characteristics of the children enrolled.

**Table 2:** Social demographic characteristics of the children enrolled in Sissa sub-county, Wakiso

Variable	School					Total
	Kitende	Bethel	Mpumudde	Jjanyi	Faith Trust	
	*N* (%)	*N* (%)	*N* (%)	*N* (%)	*N* (%)	*N* (%)
Sex:						
Male	58 (48.3)	14 (24.6)	20 (37.0)	31 (43.1)	26 (40.6)	149 (40.7)
Female	61 (51.7)	43 (75.4)	34 (63.0)	41 (56.1)	38 (59.4)	217 (59.3)
House Type^a^						
Single	72 (60.5)	35 (61.4)	44 (81.5)	44 (61.1)	49 (76.6)	244 (68)
Semi detached	44 (37.0)	18 (31.6)	10 (18.5)	28 (38.9)	15 (23.4)	115 (32)
School Location:						
Rural	0	0	54	72	0	125 (34)
Peri-urban	119	57	0	0	64	241 (66)
School Type Child Attends:						
Private	0	57	0	0	64	121(33)
Government	119	0	54	72	0	245 (67)
Class child Attends:						
Lower	45 (37.8)	21 (36.8)	27 (50.0)	45 (62.5)	30 (46.9)	168(45.9)
Middle	49 (41.2)	9 (15.8)	15 (27.8)	22 (30.6)	26 (40.6)	121(33.1)
Upper	25 (21.0)	27 (47.4)	12 (22.2)	5 (6.9)	8 (12.5)	77 (21)

^a^7 children had this information missing

### GAS carriage

There were 68/366(18.5%) positive throat swab cultures and 35/366(9.5%) positive ASOT tests. Fifty-nine (16.1%) children had carriage (a positive throat swab culture and a negative ASOT) as shown in Table 3. Of the 59 children with carriage, 23.5% were from Kitende, 29.8% from Bethel, 9.3% from Mpumudde, 9.7% from Jjanyi and 3.1% from Faith trust primary schools. Nine children, age more than 10 years, had GAS infection (positive throat culture and ASOT).

**Table 3:** A comparison of the throat swab culture and ASOT tests for children attending Primary Schools in Sissa sub-county, Wakiso

	ASOT Positive (*n* = 35) *N* (%)	ASOT Negative (*n* = 331) *N* (%)	
Throat swab culture: Positive (*n* = 68)	9 (2.5)	59 (16.1)	
Negative (*n* = 298)	26 (7.1)	272 (74.3)	

All the GAS isolates were susceptible to penicillin and vancomycin. The isolates had 80, 76, 61 and 45% susceptibility to ceftriaxone, azithromycin, erythromycin and tetracycline respectively.

### Clinical characteristics of the primary school children with GAS positive throat swab culture enrolled in Sissa sub-county Wakiso

The children who were culture positive for GAS were 68. The females were 45 (66%) and the mean age was 10.6 yrs. (SD 2.6). Table 4 shows the symptoms and physical findings. Of the 68 children with a﻿ GAS positive culture, four children had a fever, sore throat and an abnormality in the throat. One of the four children had positive results for culture and ASOT. There was no child with a documented history of RHD.

**Table 4:** Characteristics of the primary school children with GAS positive versus GAS negative throat swab culture in Sissa sub-county, Wakiso

Characteristic	GAS positive *N* (%)	GAS negative *N* (%)	OR(95% C.I)	*P* value
Sex				
Male	23 (33.8)	126(42.3)	1.43(0.83–2.49)	0.200
Female	45 (66.2)	172(57.7)		
Age				
< 10y	32(47.1)	162(54.4)	1.34(0.79–2.27)	0.277
> 10 yr	36(52.9)	136(45.6)		
History of fever				
Yes	4 (5.9)	15(5)	1.18(0.38–3.67)	0.776
No	64 (94.1)	283(95)		
Cough				
Yes	31 (45.6)	110(36.9)	1.43(0.84–2.44)	0.185
No	37 (54.4)	188(63.1)		
Sore throat				
Yes	8 (11.8)	61(20.5)	0.52(0.24–1.14)	0.098
No	60 (88.2)	237(79.5)		
Palatal petechae				
Yes	2 (2.9)	23(7.7)	0.36(0.08–1.58)	0.159
No	66 (97.1)	275(92.3)		
Tonsillar enlargement				
Yes	6(8.8)	50(16.8)	0.48(0.20–1.17)	0.100
No	62(91.2)	248(83.2)		
Cervical adenopathy				
Yes	9(13.2)	57(19.1)	0.64(0.30–1.17)	0.254
No	59 (86.8)	241(80.9)		

### Factors associated with GAS carriage

At bivariate analysis, the children from peri-urban compared to rural schools were 2 times more likely to have carriage (Table 5). Children from classes of more than 0.8 children per square meter were also 2 times more likely to have carriage.

**Table 5:** Bivariate analysis of the factors associated with GAS carriage among primary school children in Sissa sub-county, Wakiso

Variables	GAS carriage				
	No	Yes	unadjusted OR	95% C.I	*P*-value
Age greater <10 years	163 (84.0%)	31 (16.0%)			
Age greater >10 yrs	114 (83.7%)	28 (16.3%)	1.029	0.589–1.797	0.921
male	128 (85.9%)	21 (14.1%)			
Female	179 (82.5%)	38 (17.5%)	1.304	0.731–2.327	0.369
Rural school	113 (90.4%)	12 (9.6%)			
Peri urban school	194 (80.5%)	47 (19.5%)	2.270	1.156–4.457	0.017
Government school	205 (83.7%)	40 (16.3%)			
Private school	102 (84.3%)	19 (15.7%)	0.945	0.521–1.714	0.853
Household with >4 people	222 (82.8%)	46 (17.2%)			
Household with 4 or less people	84 (87.5%)	12 (12.5%)	0.693	0.35–1.371	0.292
Class with >3 windows	154 (85.6%)	26 (14.4%)			
class with 3 or less windows	153 (82.3%)	33 (17.7%)	1.269	0.725–2.223	0.404
Semi-detached house	98 (86.0%)	16 (14.0%)			
Singles house	204 (83.6%)	40 (16.4%)	1.213	0.648–2.272	0.546
Fever:					
no	290 (83.6%)	57 (16.4%)			
yes	17 (89.5%)	2 (10.5%)	0.601	0.135–2.671	0.503
Sore throat:					
no	245 (82.5%)	52 (17.5%)			
yes	62 (89.9%)	7 (10.1%)	0.534	0.231–1.233	0.142
Cough:					
no	192 (85.3%)	33 (14.7%)			
yes	115 (81.6%)	26 (18.4%)	1.322	0.753–2.323	0.331
Palatal petechiea					
no	284 (83.3%)	57 (16.7%)			
yes	23 (92.0%)	2 (8.0%)	0.435	0.1–1.896	0.268
Tonsillar enlargement:					
no	256 (82.6%)	54 (17.4%)			
Yes	51 (91.1%)	5 (8.9%)	0.467	0.178–1.224	0.121
Cervical nodes:					
no	249 (83.0%)	51 (17.0%)			
yes	58 (87.9%)	8 (12.1%)	0.676	0.304–1.502	0.337
Parent’s employment:					
informal	256 (83.9%)	49 (16.1%)			
Formal	46 (86.8%)	7 (13.2%)	0.795	0.339–1.864	0.598
Congestion:					
< 0.8children/m2	51 (91.1%)	5 (8.9%)			
> 0.8children/m2	256 (82.6%)	54 (17.4%)	2.152	0.820–5.643	0.119
BMI					
Underweight	9 (81.8%)	2 (18.2%)	1.000		
Normal	294 (83.8%)	57 (16.2)	0.872	0.184–4.144	0.864
Over weight	4 (100.0%)	0 (0.0%)	1.000		

All factors, whose *p*-value were less than 0.2, were subjected to logistic regression. Factors that were known to influence the outcome were included in the multivariate analysis even if their *p* value was more than 0.2. The only factor associated with GAS carriage was school location. The peri-urban schools were associated with GAS carriage; *p*-value was 0.049 as shown in Table 6.

**Table 6:** Multivariate analysis for the factors associated with GAS carriage among primary school children in Sissa sub-county Wakiso

	Unadjusted			Adjusted		
	OR	95% CI	*p*-value	OR	95% CI	*P*-value
Peri-urban	2.270	1.156–4.457	0.017	2.477	1.005–6.107	0.049
Sore throat	0.534	0.231–1.233	0.142	0.447	0.179–1.116	0.085
Tonsillar enlargement	0.467	0.178–1.224	0.121	0.650	0.235–1.796	0.406
Parent’s employment	0.795	0.339–1.864	0.598	0.629	0.257–1.537	0.309
Female	1.304	0.731 –2.327	0.369	1.269	0.69–2.332	0.443
Household with less than 4	0.693	0.35 –1.371	0.292	0.628	0.301–1.309	0.215
Palatal petechiea	0.467	0.178–1.224	0.121	0.593	0.129–2.725	0.502
Congestion >0.8children /m2	2.152	0.820– 5.643	0.119	1.013	0.297–3.461	0.983

## Discussion

This study describes the prevalence of GAS and factors associated with GAS pharyngeal carriage among children attending primary schools in one of the most populated districts of Uganda, Wakiso district.

The prevalence of GAS pharyngeal carriage was 16%. In the peri-urban schools, 1 in 5 children had GAS carriage. The prevalence in this study is slightly higher than the 12% pooled prevalence in a meta-analysis by Shaikh et al. [1] and that of a study done in the Ethiopian cities of Addis Ababa, Gondar and Dire Daua that was at 9.7% [9]. The prevalence is still higher than that found by Engel et al. of 3% among healthy school children in the lower socio class communities of Cape Town [10]. The high crowding index in Sissa primary schools, especially in the urban schools, is the probable cause of the high prevalence.

During the assessment for GAS carriage, 2.5% of the children had GAS infection. This proportion may be an under-estimate since most children with GAS infection are likely to be symptomatic and likely to stay at home during illness. However, a small number of children with active infection still attend school and probably do not get medical attention. In a clinic-based study in South Africa, the prevalence was 21.6% among children who presented with a history of sore throat [11]. In the current study, the children were relatively well and this explains the low prevalence of active infection as opposed to studies that have assessed symptomatic/sick children.

A few children had a positive ASOT test but a negative throat swab culture. This reflects either antecedent GAS infection or may be infection secondary to group C or G beta –hemolytic infection [4, 12]. ASOT may remain positive for several months following an initial infection [12].

Most of the children with GAS positive culture had at least a symptom or a sign. The children with GAS positive infection usually present with sudden onset of sore throat, fever, patchy tonsillopharyngeal exudates, palatal petechiae and anterior cervical adenitis (tender nodes) [13].

Two thirds of the children with symptoms were females and for this study, there were more females evaluated than males. Therefore, the high percentage of females with GAS could probably be attributed to the slightly high percentage of females in the overall sample size. In a South African study, they found equal distribution of GAS positive findings between the sexes [10].

Quite a number of children with a positive throat swab culture had a cough. Cough is not a common symptom of GAS carriage nor infection. Cough, as a symptom has been associated with viral causes in patients who also have a sore throat [13]. Since we were assessing for carriage this reflects probably carriage with inter-current viral infections in the study population.

There were quite a number of children with signs and symptoms but a negative culture. This could have been due to other non-streptococcal causes (viruses) or previous antibiotic use. Viruses are the commonest cause of pharyngitis [5]. Other bacterial causes of pharyngitis include group C and G beta-hemolytic streptococci [4].

There were a few children with sore throat, fever and tonsillar enlargement found with GAS positive culture. The majority of children did not have clinical features because children with symptoms were more likely to have stayed at home at the time of the examination. The other reason was that we were assessing relatively well children.

Asymptomatic carriage of GAS is common among contacts of patients with GAS pharyngitis [13]. Almost a third of individuals with pharyngitis that warrant testing and treatment have been in contact with a GAS carrier. In our study, all children with a positive throat culture for GAS received treatment. Most literature though does not support treatment of GAS carriers because of their low risk of transmission.

Several factors have been associated with GAS carriage among children. The factor that was associated with GAS carriage in this study was school location. Children in peri-urban schools were 2 times more likely to have GAS carriage. This finding is similar to that found in Fiji schools where GAS carriage was associated with peri-urban schools [14]. The study conducted in Fiji, had a similar population and setting as in our study.

The current ministry of education Uganda recommendation is 0.8 pupils per square meter i.e. not less than 51.04m^2^ of floor area for pupils not more than 40 per class (Uganda licensing and registration guidelines of private schools in Uganda). The crowding index in the classes was 1.3 children per classroom floor area with the peri-urban schools having a higher index compared with the rural schools.

The average household in this study was six people though we were not able to assess the floor area in the homes of the participants. Hence, we were not able to determine whether this influenced the high prevalence.

In Chennai, India among slum children aged 5–15 yrs., factors that were significantly associated with GAS isolation were the father’s occupation, number of windows on the family house and age [15]. Our study, did not find age nor guardian’s occupation to be associated with GAS carriage.

In South Africa, among the lower socioeconomic populations, carriage was neither associated with gender nor age [10]. These populations are similar to ours though the economic status may be different despite both being of low social class.

This study had several limitations. Winter and rainy seasons have high peaks of GAS colonization [16]. The study took place during a dry season and the effect of this on GAS carriage cannot be ascertained.

Sick children who did not attend school missed out and the likely probability that they did harbor the organism might have affected our findings. This may also explain why we did not find any children with history of RHD.

We were not able to determine the household floor space for the participants to determine whether this would have influenced our outcome. The participants spend their daytime both at home and school.

Anaerobic cultures may increase the proportion of positive culture results. Because of limited funds, aerobic cultures were used and these yield 90–95% positive results [13].

Recent antibiotic use was not included due to challenges of documentation and off the counter self-prescriptions in our setting. This may have affected our results.

## Conclusion

In this study, the prevalence of GAS was high with the peri-urban schools having higher rates of carriage. In Sissa sub-county Wakiso, the high crowding index in peri- urban schools may have increased the risk of GAS colonization among pupils.

## Additional file

Additional file 1: Group a streptococcus dataset Wakiso, Uganda. (XLS 133 kb)

## Abbreviations

GAS: Group a beta-hemolytic streptococcus
RF: Rheumatic fever
RHD: Rheumatic heart disease
ASOT: Anti-streptolysin O titres
BMI: Body mass index
